# Systemic tuberculosis presenting with acute transient myopia: a case report

**DOI:** 10.1186/1752-1947-2-350

**Published:** 2008-11-17

**Authors:** Sher A Aslam, Shahram Kashani, Roland K Morley

**Affiliations:** 1 Moorfields Eye Hospital, 162 City Road, London, EC1V 2PD, UK; 2 Northwick Park Hospital, Watford Road, London, HA1 3UJ, UK

## Abstract

**Introduction:**

Transient myopia has been reported to occur in a number of conditions, either ocular in origin or associated with an underlying systemic cause. We present a rare case of this abnormality occurring in the setting of systemic tuberculosis.

**Case presentation:**

A 29-year-old Indian woman presented with sudden onset blurred distance vision and fever. Examination revealed visual acuity of counting fingers in both eyes improving to 6/9 with pinhole with N5 reading acuity. Anterior segment examination revealed narrow angles on gonioscopy. Posterior segments were normal. Systemic examination revealed a fluctuant mass in her left loin, aspiration of which yielded pus which was culture-positive for *Mycobacterium tuberculosis*. The Mantoux test elicited a strongly positive reaction. Chest X-ray and magnetic resonance imaging of the brain were unremarkable. Computed tomography scan and magnetic resonance imaging of the spine and abdomen revealed a large psoas abscess communicating with the loin mass. Two vertebrae were involved but not the spinal cord or canal.

**Conclusion:**

Transient myopia is a rare presenting feature of systemic tuberculosis. A postulated mechanism in this patient is that development of a uveal effusion related to systemic tuberculosis caused anterior rotation of the iris-lens diaphragm, thereby inducing narrowing of the angle and acute myopia.

## Introduction

Transient myopia has been reported as a clinical feature related to a number of causes, including connective tissue disease [1], pharmacological effect [2], or following trauma [3]. Its pathogenesis depends on the underlying cause and can be related to corneal, lenticular, or uveal changes. We present a rare case of transient induced myopia as a presenting feature of systemic tuberculosis.

## Case presentation

A 29-year-old Indian woman presented with a sudden onset of bilateral painless, blurred distance vision. One day before this, she had developed a fever and felt generally unwell. There was no past ocular or medical history and she was taking no medication.

Examination revealed bilateral unaided visual acuity of counting fingers improving to 6/9 with pinhole. The patient reported having had unaided 6/6 visual acuity before the onset of symptoms. Near vision testing revealed unaided N5 acuity in both eyes. Extraocular movements, colour vision and pupil reactions were normal with no features of accommodative spasm, namely miosis and convergence excess. Although intraocular pressures were normal at 16 mmHg in both eyes, both anterior chambers were shallow and gonioscopy revealed narrow angles (grade 0 to 1; Shaffer method). There was no anterior uveitis and ocular media were clear. Fundal examination was unremarkable showing healthy optic discs and maculae with no evidence of chorioretinitis.

**Figure 1:**
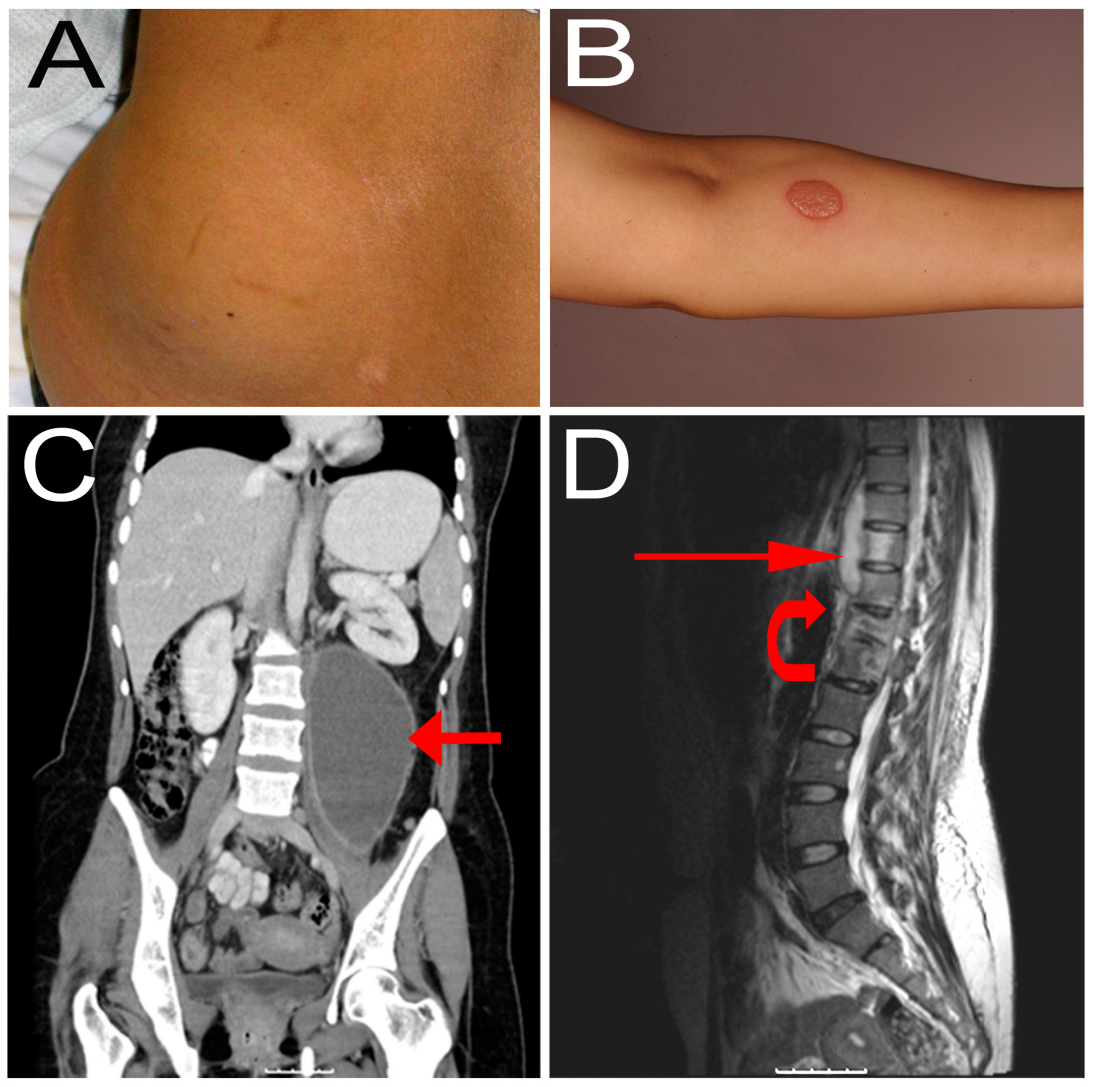
**A) Subcutaneous mass in the left loin.** B) Result of Mantoux test eliciting a strongly positive skin reaction. C) Coronal computed tomography scan with contrast showing large left psoas abscess (red arrow). D) T2-weighted sagittal magnetic resonance imaging scan showing paravertebral psoas collection (straight red arrow) and extensive involvement of T12 and L1 vertebral bodies with signal change and loss of intervertebral disc space at this level (within bounds of curved red arrow).

The only abnormalities on physical examination were pyrexia of 38.5°C, and a 10 cm × 10 cm fluctuant, non-erythematous, cold mass in the left loin (Fig. 1A). Fine needle aspiration of this lesion revealed thin, watery, purulent content which on initial microscopy revealed pus but no organisms. Staining for acid fast bacilli was negative. Debris consistent with caseation was seen on microscopy of the pus, but no granulomata were noted. *Mycobacterium tuberculosis *was eventually cultured from this aspirate 3 weeks later.

The patient had not received the bacille Calmette-Guerin (BCG) vaccination. A Mantoux test was strongly positive with a blistering skin reaction measuring 15 mm in diameter (Fig. 1B). Aside from abnormal acute phase reactants (ESR and CRP measuring 76 and 41, respectively), all other blood tests were normal, including white cell count, serum ACE, HIV and VDRL. Chest X-ray and MRI of the brain and orbits were unremarkable, the latter not revealing any gross intraocular disease.

Subsequent CT and MRI of the spine and abdomen demonstrated a large psoas abscess, measuring 14 × 9.7 × 9.5 cm (Fig. 1C). This communicated with the subcutaneous loin mass. There was also extensive vertebral involvement at T12 and L1 (Fig. 1D). Although there was slight narrowing of the spinal canal, the cord was not involved.

Her ophthalmic symptoms improved spontaneously whereby at day 3 she had no subjective complaint of visual problems and day 4, her unaided visual acuity measured 6/6 bilaterally. Examination now revealed deep anterior chambers with a bilateral, low grade anterior uveitis. Fundal examination was again unremarkable.

Given the strongly positive Mantoux reaction, the presence of caseative necrosis and the pattern of vertebral and psoas muscle involvement, a diagnosis of systemic tuberculosis was highly likely. Thus, before cultures became positive some 3 weeks later, the patient was commenced on quadruple anti-tuberculous therapy on day 6, after imaging and collection of samples were completed. A standard unsupervised oral regimen was used. This consisted of Rifinah 300 two tablets once a day (each tablet containing rifampicin 300 mg and isoniazid 150 mg), ethambutol 1 g once a day and pyrazinamide 1.5 g once a day. She tolerated this well and culture of the pus yielded *M. tuberculosis *fully sensitive to rifampicin, isoniazid, ethambutol and pyrazinamide. Thus after 2 months of quadruple therapy, her treatment was rationalised to continue with Rifinah 300 alone with the intention to treat for at least 8 months.

## Conclusion

This patient's ocular features were suggestive of acute, transient myopia. The postulated mechanism is that a ciliary body effusion, not evident on clinical examination, resulted in forward rotation of the iris-lens diaphragm, causing shallowing of the anterior chamber and a lens-induced myopic shift. Definitive proof of this would have been obtained by ultrasound biomicroscopy of the anterior chamber angle in the acute setting. Uveal effusion with induced myopia has been reported in the setting of HIV, whereby such an effusion – for which the authors were unable to explain the pathophysiology – confirmed on B-scan ultrasonography, precipitated anterior rotation of the iris-lens diaphragm precipitating angle closure glaucoma [4]. This case was successfully managed using topical steroids, aqueous suppressants and mydriatics. The latter are used in the setting of angle closure secondary to anterior rotation of the iris-lens diaphragm as they result in posterior movement of these structures, thereby opening up the angle. Pilocarpine, a miotic agent which is used in angle closure secondary to pupil block, causes further anterior rotation of the iris-lens diaphragm and is therefore contraindicated in such cases. Determining the aetiology of angle closure is therefore fundamental to administering appropriate therapy.

Ocular tuberculosis remains primarily a clinical diagnosis owing to the absence of ocular biopsy in the majority of cases. PCR techniques are available in order to demonstrate the presence of tubercule bacillus DNA in intraocular fluids [5], however, where a clinical diagnosis of ocular tuberculosis is evident, subjecting an eye with good visual acuity to intraocular biopsy carries the potentially devastating hazard of infective endophthalmitis and resultant blindness. The clinical features of ocular tuberculosis are summarised in Table 1. Ciliary body involvement in ocular tuberculosis is a well recognised entity. However, secondary angle closure has been reported to occur most commonly in the setting of anterior chamber inflammation with the formation of synechial adhesions between the iris and lens resulting in a pupil block scenario and subsequent iris bombé.

**Table 1:** Clinical features of ocular tuberculosis [5]

Adnexal	Lid mass
	Lacrimal gland mass
Anterior Segment	Conjunctival granuloma
	Scleritis or sclerokeratitis
	Phlyctenulosis
	Interstitial keratitis
	Anterior granulomatous uveitis
	Cyclitis with ciliary body granuloma
	Cataract

Posterior segment	Vitritis
	Papillitis, optic or retrobulbar neuritis
	Localised or multifocal choroiditis, chorioretinitis
	Retinal vasculitis
	Orbital granuloma

Sudden onset myopia and angle closure may be a presenting feature of systemic tuberculosis. Although the exact aetiologic and pathophysiological mechanisms in this individual are unknown, we presume the presence of uveal effusion related to the underlying condition. This case report demonstrates the need to consider a systemic cause in a febrile patient presenting with acute myopia and angle closure.

## Abbreviations

ACE: angiotensin converting enzyme; CRP: C-reactive protein; CT: computed tomography; ESR: erythrocyte sedimentation rate; HIV: human immunodeficiency virus; MRI: magnetic resonance imaging; PCR: polymerase chain reaction; VDRL: venereal disease research laboratory

## Consent

Written informed consent was obtained from the patient for publication of this case report and any accompanying images. A copy of the written consent is available for review by the Editor-in-Chief of this journal.

## Competing interests

The authors declare that they have no competing interests.

## Authors' contributions

SA and SK analysed and interpreted the patient data regarding the ophthalmic features. RM was instrumental in the medical care of the patient, and was a major contributor in writing the manuscript. All authors read and approved the final manuscript.
